# Impact of physical activity as an adjuvant treatment in the healing of venous ulcers in primary care: active legs RCT

**DOI:** 10.1186/s12912-025-04189-0

**Published:** 2025-12-10

**Authors:** Borja Herraiz-Ahijado, Carmen Folguera-Álvarez, Ricardo Rodríguez-Barrientos, Raquel Sánchez-Ruano, Marcos Pascual-García, Pilar Mori-Vara, José Verdú-Soriano, Milagros Rico-Blázquez, Active Legs Group

**Affiliations:** 1 Dos de Mayo Healthcare Center. Primary Care Assistance Management, Madrid Health Service, Madrid, Spain; 2 Research Network on Chronicity, Primary Care and Health Promotion (RICORS-RICAPPS). Instituto de Salud Carlos III, Madrid, Spain; 3 Gregorio Marañon Health Research Institute, Madrid Health Service, Madrid, Spain; 4 Nursing Department. Faculty of Nursing, Physiotherapy and Podiatry, Universidad Complutense de Madrid, Madrid, Spain; 5 Doctoral Program in “Cuidados en Salud”, Universidad Complutense de Madrid, Madrid, Spain; 6 La Paz Healthcare Center. Primary Care Assistance Management, Madrid Health Service, Rivas-Vaciamadrid, Spain; 7 Research Unit. Primary Care Assistance Management, Madrid Health Service, Madrid, Spain; 8 Research Group on Public Health - Lifestyles, nursing methodology and carecommunity environment, Universidad Complutense de Madrid, Madrid, Spain; 9 Health Innovation Research Group. Nursing Department. Faculty of Nursing, Physiotherapy and Podiatry. Universidad Complutense de Madrid, Madrid, Spain; 10 Departamento de Enfermería Comunitaria, Medicina Preventiva, Salud Pública e Historia de la Ciencia, Facultad de Ciencias de la Salud, Universidad de Alicante, San Vicente del Raspeig, Alicante España; 11 Alpes Healthcare Center. Primary Care Assistance Management, Madrid Health Service, Madrid, Spain; 12 Centro de Salud Dos de Mayo. Gerencia Asistencial de Atención Primaria, Servicio Madrileño de Salud, Calle Coronel de Palma 1, Móstoles, Madrid 28934 Spain

**Keywords:** Varicose ulcer, RCT, Complete healing, Exercise, Nursing, Primary health care, Quality of life, Ageing

## Abstract

**Background:**

Venous ulcers negatively affect quality of life and generate high health care costs. Physical activity may improve their evolution; however, the evidence is limited and heterogeneous.

**Objective:**

To evaluate the effectiveness of a structured physical activity intervention as an adjunct treatment for the complete healing of venous ulcers in primary care at 3 and 6 months of follow-up.

**Design:**

This was a randomized, pragmatic clinical trial with 6 months of follow-up.

**Methods:**

Between February 2021 and June 2023, 44 people with a diagnosis of venous ulcers and an ankle-brachial index between 0.8 and 1.3 were recruited from 13 health centres in Madrid. Both groups received standard treatment. The intervention group also received a structured educational intervention of physical exercise and daily walking guidelines. The main outcomes were complete healing (RESVECH 2.0 scale) and time to healing (days). The secondary variables included degree of healing, ulcer area, pain, adherence, and variables related to healing and prognosis. Data were collected at the beginning and at 3 and 6 months of follow-up. Survival analysis (Kaplan‒Meier and Cox) was performed to measure the effectiveness of the treatments, as was intention-to-treat analysis.

**Results:**

At 3 months, 77.3% [95% CI 54–91] of the patients in the intervention group and 68.2% [95% CI 45–85] of those in the control group achieved complete healing, without statistically significant differences between groups. Overall adherence to the intervention was low; only 20% and 46% of the participants reached the level of compliance established in the first two visits, which progressively decreased.

**Conclusions:**

The Active Legs programme showed a positive effect on the healing of venous ulcers in primary care.

**Trial registration:**

http://NCT04039789. [https://ClinicalTrials.gov]. 11/07/2019

## Background

Venous ulcers (VUs) are lesions with substance loss between the knee and the ankle secondary to chronic venous insufficiency [1–3], with a tendency towards torpid evolution, a slight tendency towards spontaneous healing [4] and a high recurrence rate [5].

Currently, VUs represent between 75 and 80% of all lower limb (LL) ulcers [6]. In Spain, the prevalence of VUs is between 0.5% and 0.8% in the population, tripling to 3–5% in people over 65 years of age [6]. Within the primary care setting, VUs account for 2.5% of the consultations, and more than 80% of patients receive treatment at this level of care, which is highly important in the treatment and diagnosis of this pathology [7, 8]. In a 2007 study in Spain [9], the costs of dressing changes, infection episodes and hospital stays were collected, and the total cost of chronic wounds was estimated to account for between 1.5 and 3% of all health care costs.

VUs cause pain, bad odour, abundant exudate and sometimes infection, which can lead to mobility problems, sleep disorders, loss of vitality and functional dependence, negatively affecting quality of life and emotional well-being [10–12].

The usual care of VUs includes cleaning, debridement, control of exudate with dressings [5, 13, 14] and via multi-component compression therapy for the control of chronic venous insufficiency [5, 6, 15–17].

Recent studies have indicated that LL muscle pump dysfunction [3, 10, 18–21] and reduced ankle range of motion contribute to the low healing rate in these patients [22, 23]. In addition, an insufficient level of physical activity has been observed in this population [24].

Evidence suggests that daily walking and physical exercise programs, in combination with compression therapy, improve muscle pump function [25–28], healing rates [3, 10, 18–21, 29–31], quality of life [3, 21, 29] and ankle joint mobility [22, 23]. However, these studies have methodological limitations, such as small sample sizes, reduced follow-ups and great variability in the measurement instruments used.

Four recent systematic reviews have recommended the incorporation of physical exercise as a complement to compression therapy to improve healing, muscle strength, mobility and adherence to treatment [32–35].

The exercise programmes described include plantar flexion–extension, ankle twists, knee flexion–extension exercises, resistance exercises and ambulation, with face-to-face, home or combined modalities, some with technological support [3, 10, 18–23, 25–31]. Additionally, the use of pedometers has been shown to be helpful in promoting physical activity through personalized goals and ongoing feedback [36, 37].

We did not find studies in Spain that evaluated the effect of physical exercise on VU healing in primary care.

## Methods

### Objectives

To evaluate the effectiveness of a structured physical activity intervention (the Active Legs programme) as an adjunct treatment for the complete healing of VUs in primary care at 3 and 6 months of follow-up. As secondary objectives, the effects of the intervention on the rate of complete healing, the degree of healing and adherence to the intervention at 3 and 6 months of follow-up were analysed.

## Design and setting

This was a randomized, multicentre, pragmatic, open clinical trial with two parallel groups and 6 months of follow-up. The CONSORT checklist is available as supporting information (Additional file 1). The study methodology is presented in more detail in the published protocol [38]. The results presented in this article are derived from the registered clinical trial (NCT04039789) and the previously published protocol [38].

## Setting and study population

The study was conducted in 13 primary care health centres in the Madrid region. People aged 18 years or older with a diagnosis of VUs recorded in electronic medical records and an ankle brachial index (ABI) between 0.8 and 1.3 and who were undergoing follow-up in a primary care nurse consultation and signed an informed consent form were included.

People with arteriovenous ulcers, acute phase deep vein thrombosis, decompensated heart failure, acute dermatitis, rheumatoid arthritis, and antineoplastic treatment or those with absolute contraindications to physical exercise were excluded.

**Fig. 1:**
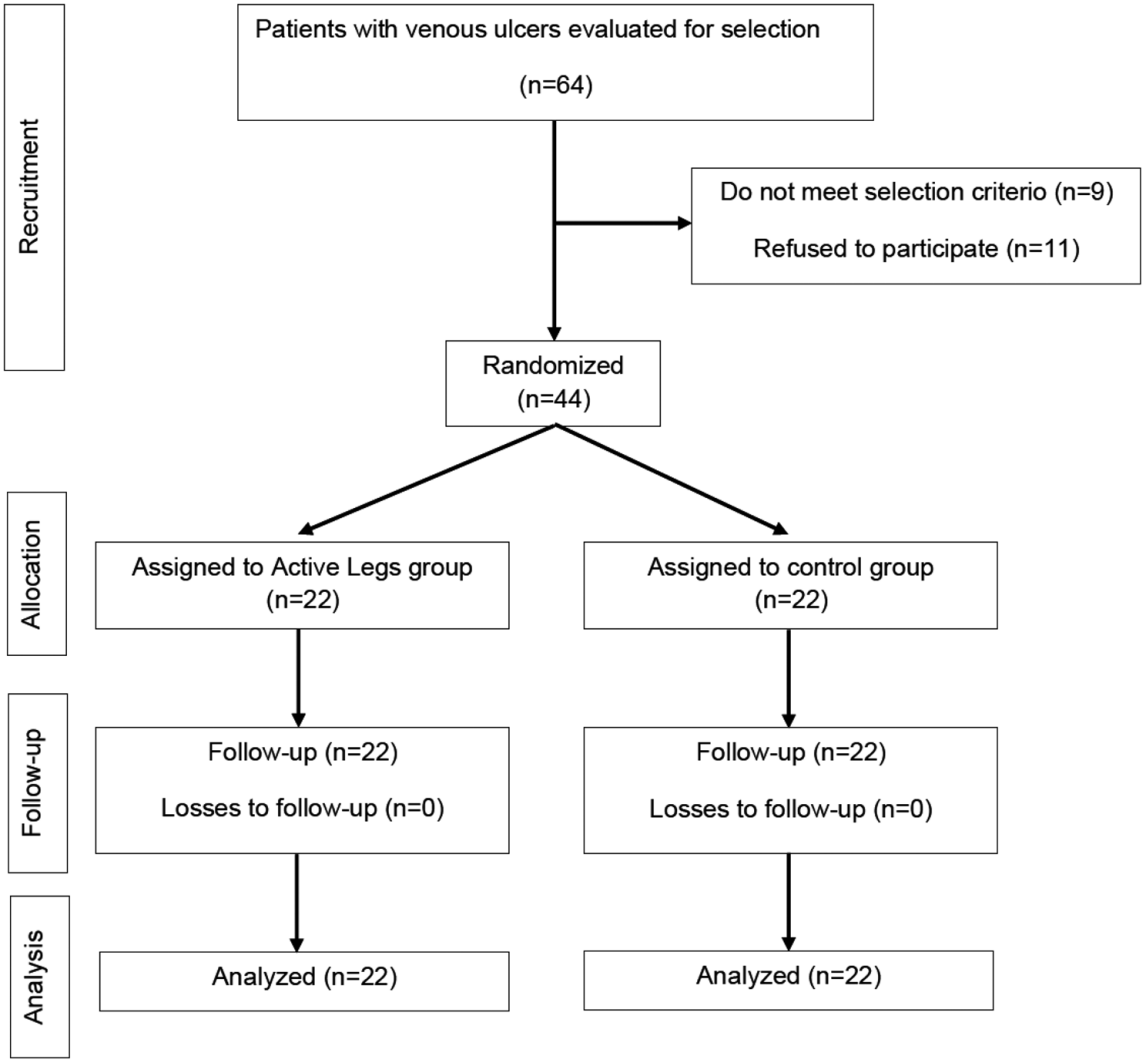
Participant recruitment flow chart

Between February 2021 and June 2023, 21 nurses who participated voluntarily in the study, recommended 64 people for participation in the study. Nine were excluded because they did not meet the inclusion criteria, and 11 refused to participate. Ultimately, 44 people were included and randomized into two groups (22 per branch). Figure 1 shows a flowchart of participant recruitment.

## Randomization and blinding

Nurses consecutively recruited the participants during consultations and requested their written informed consent before being included in the study. Randomization was carried out by means of simple randomization, generated automatically by the Electronic Data Collection Notebook (CRDe).

Given the type of intervention, it was not possible to blind either participants or professionals. However, the research team that performed the analysis was unaware of the group assignments.

## Sample size

The study was designed with a power of 80% and an alpha error of 0.05 to detect a minimum difference of 20% in people with complete healing at 3 months of follow-up. The calculated size was 224 people (112 per group), assuming a 20% loss to follow-up. [3, 25, 29]

Given the final sample size achieved, the effect size was calculated as log(HR) = 0.177 with a standard error of 0.412, and a post hoc statistical power of 29.8%. Although the hazard ratio of 1.194 aligns with the hypothesized direction of the effect, the statistical power is insufficient to reliably detect moderate effects, which supports a cautious interpretation of the results.

## Interventions

In both groups, nurses performed the usual venous wound care, following the recommendations for the treatment of skin ulcers of the Madrid Health Service [2]. The care consisted of assessment, cleaning, antisepsis, debridement, treatment in a humid environment with dressings and multicomponent compression therapy.

The intervention group received, in addition to the usual treatment, information about “Active Legs”, a structured physical activity programme designed as an adjuvant to the usual treatment.

This intervention was implemented as planned in the published protocol [38] and was introduced to participants by nurses using health education techniques. It included four progressive lower limb exercises to be performed twice daily, at least five days per week, at home. Patients received individualized health education from their nurse during the baseline consultation, covering the “Active Legs” exercise guidelines, correct execution, and recommended frequency, as well as during follow-up visits, where adherence was reinforced through theoretical and practical sessions.

Progressive daily ambulation was also recommended until the goal of 30 minutes a day, five days a week, was reached[39].

The participants received a printed “guide of recommendations for patients with venous ulcers of the Community of Madrid”, and the people in the intervention group also received a Yamax^TM^ PZ270 pedometer and an activity diary with activity guidelines and self-registration tables.

All the nurses received training in good practices and 2-hour training in Active Legs prior to the start of the study.

The intervention is described graphically and in detail in the published protocol [38].

## Data collection and outcomes

The main outcome was complete ulcer healing at 3 months (yes/no), which was considered total epithelialization maintained for at least 2 weeks. The time elapsed between the beginning of the study and complete healing (in days) was also recorded. The secondary variables included complete healing at 6 months (yes/no), degree of healing (Resvech 2.0, it was validated in Spanish by Restrepo JC in 2010. Its internal consistency showed a Cronbach’s alpha of 0.72 based on 44 standardized items, and it demonstrated content, criterion, and construct validity [40]), ulcer area (cm^2^) measured by digital photography [41], pain, prognostic and sociodemographic variables and adherence to the intervention. Adherence was evaluated in the intervention group by self-registration and pedometry, following the CONSORT recommendations for complex interventions.

The information was recorded in an electronic data collection notebook at the beginning of the study, every 15 days during the first 3 months, at the time of healing (if it occurred) and at the end of the 6-month follow-up.

## Statistical analysis

A descriptive analysis (means, medians, and distribution frequencies) was performed. A comparative analysis of the two groups was performed at the baseline visit using the appropriate tests (chi-square for comparison of proportions and Student’s t-test for comparison of means). The results of the primary outcomes were analysed on an intention-to-treat (ITT) basis. To analyse the main effectiveness, the incidence rates of ulcers with complete healing were compared and expressed as hazard ratios and 95% confidence intervals. The time to complete healing was compared using Kaplan‒Meier curves (log-rank test). To adjust for prognostic factors, Cox regression was performed. To analyse the secondary results, an explanatory model was adjusted with linear or logistic regression. Significance was set at *p* < 0.05. All analyses were performed using STATA software, version 18 [42].

## Results

During the recruitment period, 64 individuals were invited to participate in the study, of whom 11 declined due to lack of time and 9 did not meet one or more of the inclusion criteria. 44 participants were included, with 22 in each group. No participants were lost to follow-up (Fig. 1).

Baseline characteristics were similar between the groups. The mean age was 72.8 years (13.6) in the intervention group and 71.2 years (15.1) in the control group. The sex distributions was similar between groups. The majority of participants were retirees or housewives, with a predominance of individuals with low educational levels. Hypertension was the most frequent comorbidity. No statistically significant baseline differences were observed between the groups.

At the beginning of the study, the mean duration of ulcer development was 44.5 days (45) in the intervention group and 56.6 days (42.2) in the control group. In both groups, 81.8% of the participants received multicomponent compression therapy as part of the usual treatment (Table 1).

**Table 1:** Baseline characteristics of the participants in the study (*n* = 44)

	Intervention group (*n* = 22)	Control group (*n* = 22)	p
**Sociodemographic variables**			
Sex (male)	10 (43.5%)	13 (56.5%)	0.56
Age (years)	72.8 (13.6)	71.2 (15.1)	0.71
**Level of education**			0.15
* High*	3 (13.6%)	0	
* Medium*	3 (13.6%)	8 (36.4%)	
* Low*	14 (63.6%)	13 (59.1%)	
* No formal education*	2 (9.1%)	1 (4.5%)	
**Occupation**			0.91
*** Retired***	10 (45.5%)	9 (40.9%)	
*** Housewives***	6 (27.3%)	6 (27.3%)	
*Live alone*	4 (18.2%)	8 (36.4%)	0.31
**Habits and clinical variables**			
**Alcohol consumption**	4 (18.2%)	6 (27.3%)	0.72
**Tobacco use**	4 (18.2%)	4 (18.2%)	1
**Independent ambulation**	18 (81.8%)	17 (77.3%)	0.50
**VREM**			0.30
* Sedentary*	3 (13.6%)	6 (27.3%)	
* Moderately active*	6 (27.3%)	5 (22.7%)	
* Assets*	4 (18.2%)	7 (31.8%)	
* Very Active*	9 (40.9%)	4 (18.2%)	
Mean BMI	30.2 (6.5)	32.5 (8.8)	0.32
**Associated pathologies**			
Hypertension	15 (68.2%)	16 (72.7%)	1
Varicose veins	13 (59.1%)	11 (50%)	0.76
Osteoarthritis	6 (27.3%)	1 (4.5%)	0.09
Renal insufficiency	2 (9.1%)	3 (13.6%)	1
Heart failure	1 (4.5%)	5 (22.7%)	0.2
Diabetes mellitus	1 (4.5%)	4 (18.2%)	0.34
DVT background	4 (18.2%)	0	0.1
COPD	1 (4.5%)	0	1
Peripheral arterial vascular disease	1 (4.5%)	1 (4.5%)	1
**Ulcer-related variables**			
Recurrent ulcer	8 (36.4%)	9 (40.9%)	1
**Ulcer onset**			0.76
Traumatic	10 (43.5%)	8 (36.4%)	
Spontaneous	12 (54.5%)	14 (63.6%)	
Number of ulcers	2 (1.6)	1.18 (0.4)	0.17
Average time (days)	44.5 (45)	56.6 (42.2)	0.36
Resvech score 2.0	10.5 (3.2)	11.23 (3.1)	0.45
Multilayer compression therapy	18 (81.8%)	18 (81.8%)	1
**Pain**			0.7
* No pain*	2 (9.1%)	3 (13.6%)	
* Mild*	7 (31.8%)	9 (40.9%)	
* Moderate*	12 (54.5%)	8 (36.4%)	
* Intense*	1 (4.5%)	2 (9.1%)	

Data are presented as n (%) or mean (SD)

Chi-square for comparison of proportions and Student’s t-test for comparison of means

According to the Resvech 2.0 scale, at the baseline assessment, most of the ulcers were smaller than 4 cm^2^ (63.6% in the intervention group and 72.7% in the control group). The predominant involvement was in the dermis and epidermis (77.8% and 86.4%, respectively).

100% of the participants in the intervention group and 95.5% of those in the control group had necrotic and/or slough tissue in the wound bed.

A total of 45.5% of the ulcers in the intervention group and 54.5% in the control group were stagnant.

Concerning the Resvech 2.0 scale, no significant differences were observed between the groups in any of the variables evaluated (Table 2).

**Table 2:** Baseline characteristics according to the aspects evaluated in the resvech 2.0 questionnaire (*n* = 44)

	Intervention group (*n* = 22)	Control group (*n* = 22)	p
**Lesion dimensions**			0.79
< 4 cm^2^	14 (63.6%)	16 (72.7%)	
4–15 cm^2^	7 (31.8%)	5 (22.7%)	
16–35 cm^2^	1 (4.5%)	1 (4.5%)	
36–63 cm^2^	0	0	
64–100 cm^2^	0	0	
> 100 cm^2^	0	0	
**Depth/Tissues affected**			0.35
Dermis-epidermis	17 (77.8%)	19 (86.4%)	
Subcutaneous tissue	5 (22.7%)	3 (13.6%)	
Thigh	0	0	
**Edges**			0.5
Not distinguishable	0	1 (4.5%)	
Diffuse	6 (27.8%)	3 (13.6%)	
Delimited	13 (59.1%)	16 (72.7%)	
Damaged	2 (9.1%)	2 (9.1%)	
Thickened	1 (4.5%)	0	
**Type of tissue in the bed **			0.5
Necrotic	0	0	
Necrotic and/or slough	22 (100%)	21 (95.5%)	
Granulation	0	1 (4.5%)	
Epithelial	0	0	
**Exudate**			0.86
Dry	0	0	
Humid	15 (68.2%)	14 (63.6%)	
Wet	6 (27.8%)	3 (13.6%)	
Saturated	1 (4.5%)	1 (4.5%)	
With exudate leakage	0	4 (18.2%)	
**Infection/inflammation**			
Increasing pain	8 (36.4%)	4 (18.2%)	0.15
Erythema in the perilesion	13 (59.1%)	13 (59.1%)	0.62
Oedema in the perilesion	9 (41%)	9 (41%)	0.62
Temperature increase	2 (9.1%)	3 (13.6%)	0.5
Increasing exudate	4 (18.2%)	4 (18.2%)	0.65
Purulent exudate	1 (4.5%)	0	0.5
Friable fabric	5 (22.7%)	5 (22.7%)	0.64
Stagnant wound	10 (45.5%)	12 (54.5%)	0.38
Biofilm compatible fabric	8 (36.4%)	7 (31.8%)	0.5
Odour	0	2 (9.1%)	0.24
Hypergranulation	1 (4.5%)	2 (9.1%)	0.5
Increased wound size	2 (9.1%)	4 (18.2%)	0.33
Satellite injuries	6 (27.8%)	10 (45.5%)	0.17
Paleness of the tissue	2 (9.1%)	2 (9.1%)	0.69

Data are presented as n (%). Chi-square for comparison of proportions

**Fig. 2:**
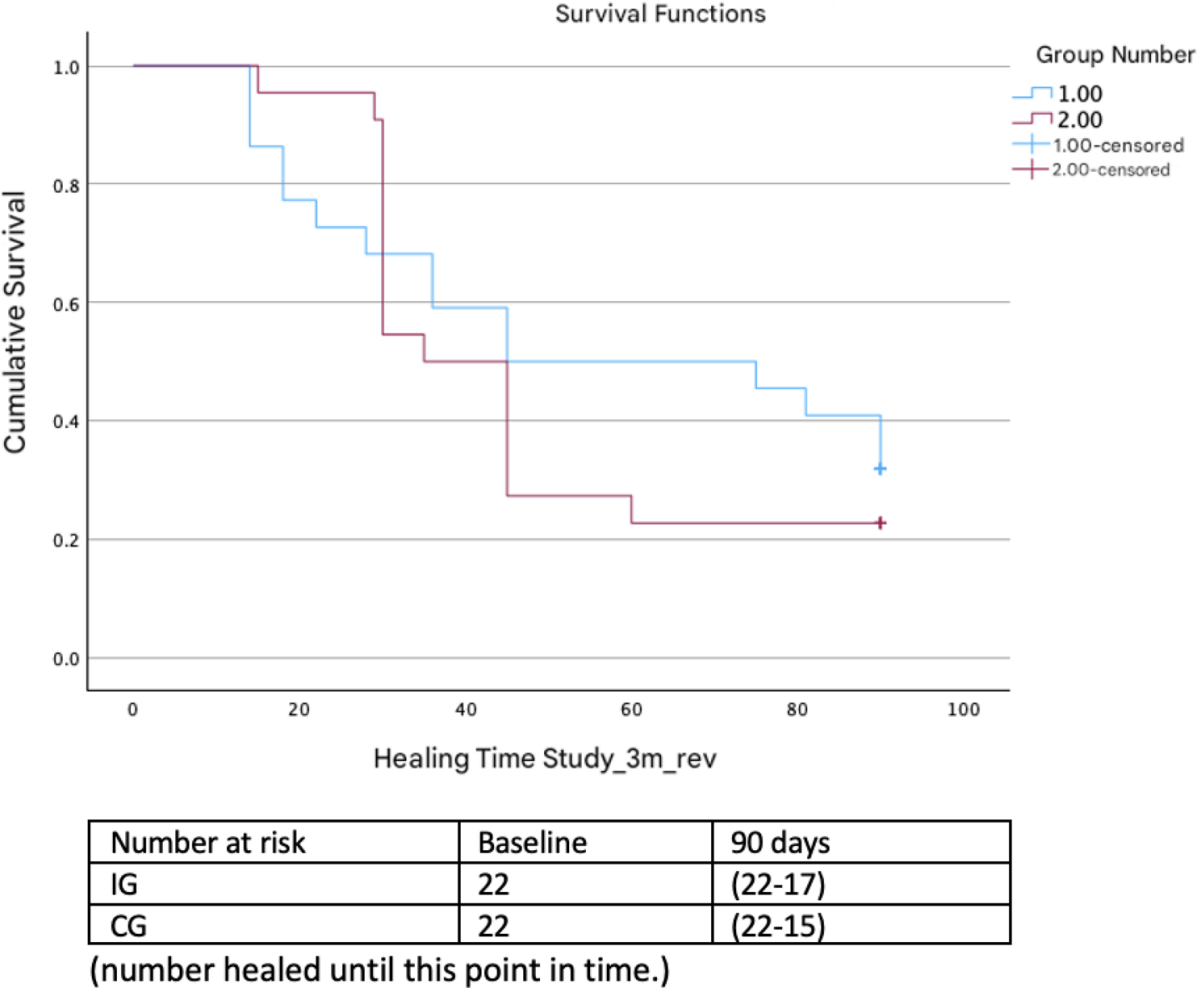
Kaplan‒Meier curves for the 3-month follow-up data

At 3 months of follow-up, 72.7% [95% CI (57–84)] of the participants achieved complete VU healing, i.e., 77.3% [95% CI (54–91)] in the intervention group and 68.2% [95% CI (45–85)] in the control group, HR = 1.20 [95% CI (0.59–2.42)] (*p* = 0.61) (Fig. 2).

The median time to healing was 45 days [95% CI (37–52)] in the total sample. In the intervention group, the median duration was 35 days [95% CI (29–40)], and in the control group, 45 days [95% CI (21–112)], with no significant differences between the groups (log rank *p* = 0.585).

**Fig. 3:**
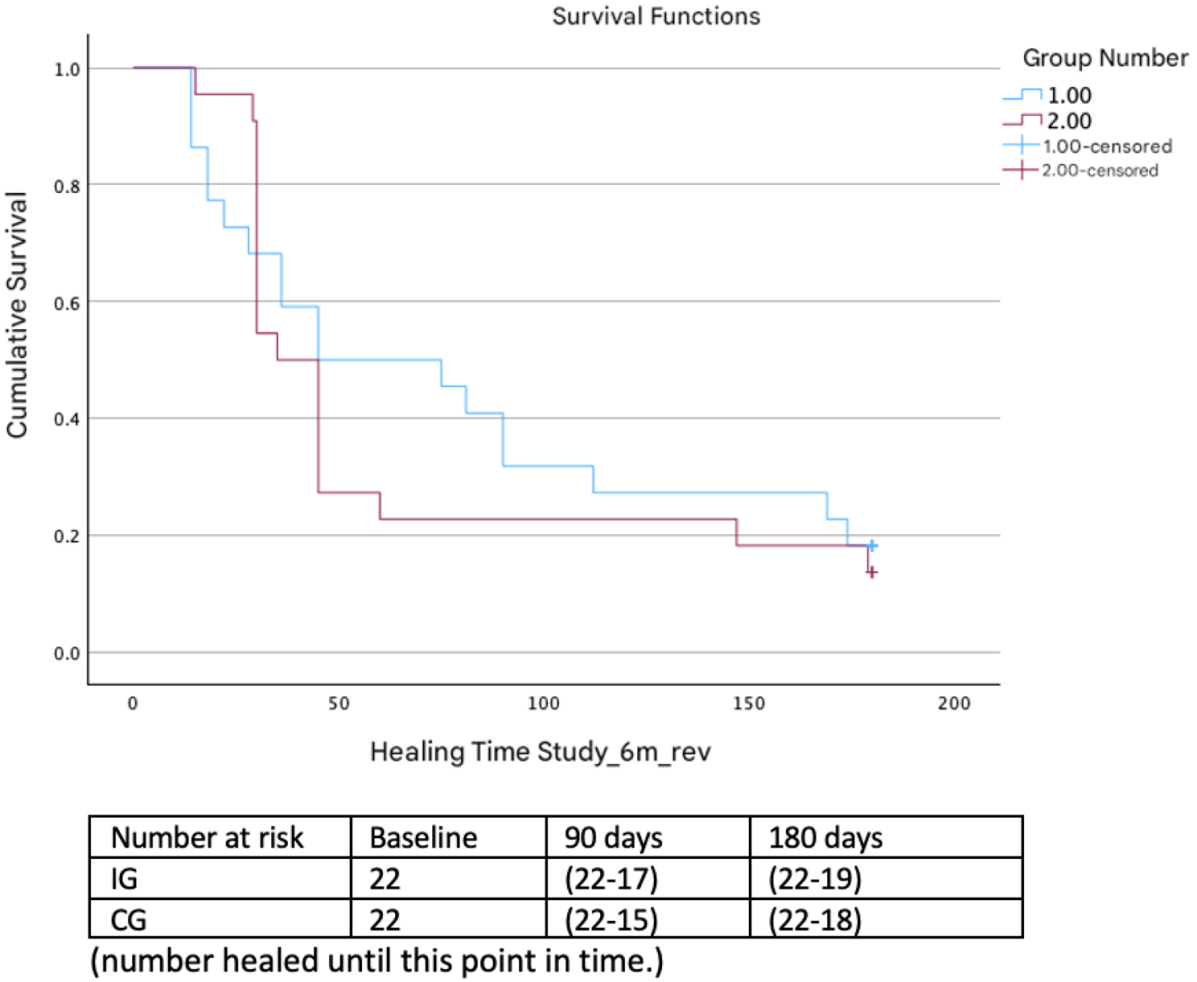
Kaplan‒Meier curves for the 6-month follow-up data

At 6 months, 84.1% [95% CI (69–92)] of the participants had compte healing, i.e., 86.4% [95% CI (64–96)] in the intervention group and 81.8% [95% CI (59–94)] in the control group, HR = 1.14 [95% CI (0.59–2.19)] (*p* = 0.68) (Fig. 3).

The median time to complete healing at 6 months was unchanged from the analysis at 3 months, with no significant differences between groups (log rank *p* = 0.670).

A Cox regression model was used to estimate the relationships of number of previous days of ulcer evolution, body mass index and baseline exercise, as measured using the VREM, with complete healing. No significant relationship was found with complete healing either at 3 months (Table 3) or at 6 months (Table 4).

**Table 3:** Cox model adjusted by activity level in categories, previous days of ulcer evolution and BMI at the 3-month follow-up

	HR	95% CI for HR Lower Upper		p
Group	1.192	0.532	2.670	0.669
**Sedentary**				0.886
**Moderately active**	0.685	0.205	2.284	0.380
**Active**	0.928	0.343	2.516	0.884
**Very active**	1.037	0.369	2.913	0.944
**Previous days of ulcer evolution**	0.994	0.985	1.003	0.207
**BMI**	1.011	0.966	1.058	0.642

**Table 4:** Cox model at 6 months adjusted for activity level in categories, previous days of ulcer evolution and BMI

	HR	95% CI for HR Lower Upper		p
Group	1.141	0.535	2.431	0.733
**Sedentary**				0.848
**Moderately active**	0.698	0.234	2.083	0.519
**Active**	0.688	0.259	1.832	0.454
**Very active**	0.899	0.340	2.382	0.831
**Previous days of ulcer evolution**	0.995	0.987	1.003	0.212
**BMI**	1.007	0.966	1.051	0.731

At three months, the degree of healing, as measured with the Resvech 2.0 scale, was reduced by an average of 4.37 points [95% CI (2,284–6,468)], and at 6 months, it was reduced by 6.41 points [95% CI (3,486–9,326)] (Table 5). When the total sample was considered, all the changes from baseline were statistically significant, but there were no differences between the groups.

**Table 5:** Estimated mean reduction (95% CI) in the resvech 2.0 scale from baseline

	Control (n = 22)	Intervention Total (n = 22)	
**15 days**	4.435 (2.223–6.646)	2.864 (0.903- 4.824)	3.547 (2.085- 5.008)
**30 days**	5.321 (2.895–7.747)	6.199 (4.261- 8.137)	5.947 (4.426- 7.467)
**45 days**	5.464 (1.996–8.932)	6.412 (3.290- 9.535)	6.035 (3.746- 8.324)
**60 days**	4.056 (0.76- 7.351)	4.289 (0.441- 8.136)	4.097 (1.715- 6.480)
**75 days**	3.611 (0.895- 6.328)	2.188 (−1.070- 5.445)	2.979 (1.003–4.955)
**90 days**	5.717 (2.788- 8.645)	2.845 (−0.190–5.880)	4.376 (2.284- 6.468)
**180 days**	5.784 (1.169- 10.398)	6.849 (2.685- 11.014)	6.406 (3.486- 9.326)

In the intervention group, the level of adherence to LL home exercises was high at the first visit. At the first follow-up visit, 54.5% of the participants reported excellent adherence (performance of more than 75% of the prescribed home exercises), 27.3% reported good adherence (50–74%), and 18.2% reported moderate adherence (25–49%).

**Fig. 4:**
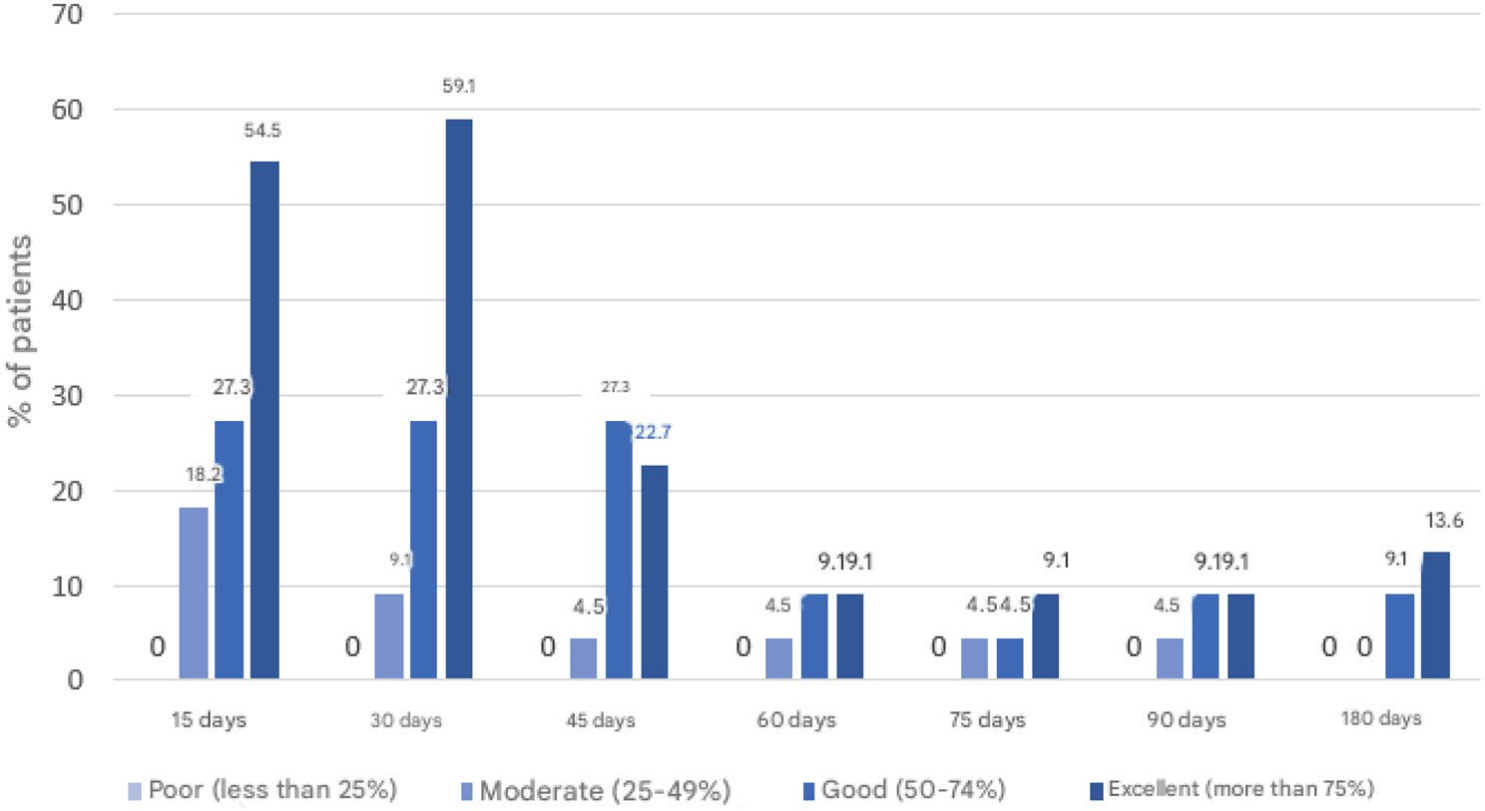
Percentage of participants by visit and level of compliance with home exercises

This pattern was maintained at the second visit, but from the third visit, a progressive decrease was observed, with excellent adherence decreasing to 22.7% and stabilizing between 9 and 13% at subsequent visits (Fig. 4).

Table 6 presents the median and interquartile range of the daily step count in the intervention group during each follow-up visit. The median ranged between approximately 2500 and 3500 daily steps.

**Table 6:** Median and interquartile range of the mean number of steps

	Median	Interquartile range (IR)
Visit 1 (15 days)	2781.5	5810
Visit 2 (1 month)	3246	3559
Visit 3 (1 month and 15 days)	2017	3606
Visit 4 (2 months)	3523	4967
Visit 5 (2 months and 15 days)	3762	5743
Visit 6 (3 months)	3859.5	6939
Visit 7 (6 months)	3335.5	4597

Overall adherence to the intervention, defined as performing more than 75% of the home exercises and a daily average of more than 3000 steps, was generally low; in the first two visits, the estimated percentages of participants with global adherence were 20 and 46%, respectively.

**Fig. 5:**
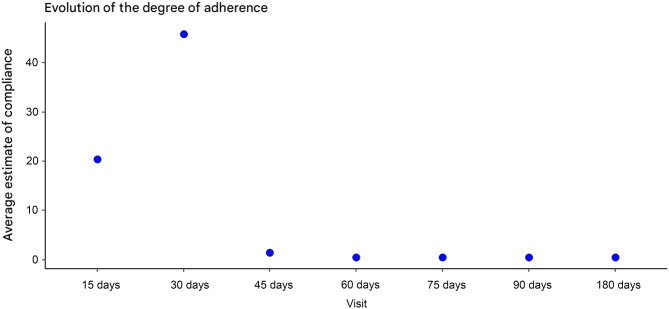
Estimation of the mean percentage of participants with global adherence to treatment until healing

After the third visit, a marked decrease was observed, stabilizing at levels below 15% for subsequent visits (Fig. 5).

When comparing the people in the intervention group who met the global adherence criteria of the intervention with those in the control group, no significant differences were observed in effectiveness at 3 months of follow-up, according to the log rank test results (*p* = 0.64).

## Discussion

This study evaluated the effectiveness of a structured programme involving home physical exercise and daily walking, i.e. “Active Legs,” as an adjunctive treatment for the healing of VUs in primary care. Although a greater proportion of complete healing was observed in the intervention group both at 3 months (77.3% vs. 68.2%) and at 6 months (86.4% vs. 81.8%), the differences did not reach statistical significance. These results are consistent with the findings of other clinical trials that, despite showing favourable trends, failed to show significant differences [18–20, 30].

However, some studies with larger sample sizes or longer durations of follow-up reported significant differences. For example, Heinen [10] reported significantly shorter healing times after 18 months of follow-up with an intervention based on walking and LL exercises; O’Brien [3] also reported significant differences in healing rates at 3 months, but only in participants with high adherence to the exercise program.

Several factors may account for the findings observed in this study. First, the small sample size limited the statistical power, making it difficult to detect significant differences between groups. Second, most of the ulcers included were “simple” in nature, with a mean RESVECH 2.0 score of 10.8 points and a median ulcer area of 2.54 cm^2^, which likely favored spontaneous healing in both groups and may have attenuated the differential effect of the intervention. This type of lesion also represents the most common profile of venous ulcers managed in primary care settings, as reported in previous studies [43, 44].

Third, variability in the application of topical treatment and compression therapy among healthcare professionals, despite the training provided, may have influenced the results. Nevertheless, heterogeneity in the use of wound care products was minimal, as the Primary Care Management of Madrid implemented in 2010 the *Improvement Plan for the Care of Patients with Ulcers*, which included specific professional training, the development of a *Guideline of Recommendations* [2], and the establishment of a centralized procurement procedure. Consequently, the availability and use of wound care products, as well as the implementation of multicomponent compression therapy, were homogeneous and standardized across participating centers.

Although multicomponent compression therapy is currently considered the gold standard for the management of chronic venous disease [17, 43–47], its use in Spanish primary care remains limited.

Although no differences were observed in healing, other studies have reported benefits of physical exercise on the function of muscle pumps in the leg, such as an increase in the ejection fraction and a reduction in the residual fraction [26, 28]. These findings suggest that exercise may have beneficial physiological effects even if it does not directly translate into a higher rate of healing in the short term.

Regarding adherence, the results indicated moderate compliance with the home-based exercise program: between 54 and 60% of participants completed more than 75% of the prescribed exercises during the first two visits, with adherence declining after six weeks, partly due to early healing among the most adherent patients. These findings are consistent with those reported by O’Brien [3, 25], who observed 59% adherence, and Heinen [10], who achieved 77% adherence with motivational support, which subsequently decreased to 59% at 18 months. Studies incorporating in-person or online supervision by physiotherapists [20, 28] or nurses [48] reported higher adherence levels (71–81%), highlighting the importance of professional guidance in sustaining patient engagement.

Daily walking was the component with the lowest adherence, with a median of 3498 steps per day. Similar results were reported by Heinen [10], with participants averaging only 10 minutes of walking per day, and by Meagher [24], where only 33% achieved 10,000 daily steps. These low levels of ambulation are consistent with the literature describing the sedentary lifestyle common among patients with venous ulcers [5, 49]. Overall adherence to the program—defined as ≥75% completion of exercises and ≥3,000 steps per day—ranged from 20% to 46% during the first two visits, with a marked decline after six weeks. Approximately half of the participants who adhered to the home exercises did not meet the daily step target, a finding also reported by Heinen [10].

## Limitations and strengths

One of the main limitations of this study was that it did not reach the expected sample size (*n* = 224), which reduced the statistical power to detect significant differences. Recruitment was affected by external factors such as the COVID-19 pandemic, the consequent reorganization of primary care and the labour mobility of professionals to achieve health care objectives, which relegated research activity to the background.

Another relevant limitation was the low overall adherence to the intervention, especially with regard to daily ambulation. Although more than 50% of the participants in the intervention group reported performing > 75% of the home exercises at the first visit, only between 20 and 46% reached the goal of daily steps ( > 3000), and this proportion decreased dramatically after 6 weeks.

Considering the different possible ways of local wound treatment and compression therapy, there is substantial potential for variation in these interventions. Certainly, not every case was treated exactly the same, so differences in any of these treatments could also explain variations in patient outcomes.

Furthermore, the biweekly self-reported assessment of adherence to the home exercise programme, necessitated by the inability of nursing staff to conduct daily monitoring, may have introduced bias into the results. Because adherence was self-reported, actual adherence could have been lower than reported due to social desirability or overconfidence biases. This reduced adherence may partly account for the smaller-than-expected effects of the intervention on wound-healing rates. Nevertheless, the use of pedometers provided an objective measure of daily ambulation, helping to mitigate some of these limitations.

In terms of strengths, there were no dropouts during follow-up, which reinforces the viability of the programme in primary care. In addition, standardized and validated tools such as the RESVECH 2.0 scale and ABI measurements were used to ensure sample homogeneity. The follow-up was intensive and was carried out both in person and by telephone, which allowed more robust data collection.

## Applicability and future research perspectives

This study highlights the importance of a comprehensive approach to the management of patients with VUs, taking into account the characteristics of each lesion and the training of primary care nursing staff to optimize healing and treatment adherence. Strategies such as supervised exercise programs, self-care education, reinforcement of compression therapy, and the use of objective monitoring tools (e.g., RESVECH 2.0, pedometers) enable more precise follow-up and enhance patient motivation.Therefore, it is considered essential to strengthen in-person prescription and follow-up by healthcare professionals who care for this population, complement these actions with digital monitoring tools (such as apps with feedback features), and promote outdoor or community-based activities adapted to patient preferences, in order to improve adherence to daily walking routines.

Particularly, it is essential to implement targeted reinforcement strategies during follow-up visits for patients with low adherence to the intervention. Reinforcement should be delivered in an individualized manner, tailored to each patient’s adherence level at every follow-up visit, in order to maximize engagement, promote behavioral change, and optimize clinical outcomes.

Future research should include larger sample sizes, standardized exercise protocols, and long-term follow-up to assess the effectiveness and clinical applicability of these interventions. Furthermore, studies with greater statistical power are needed to confirm non-significant trends observed in smaller trials.

Overall, this study reinforces the need for an evidence-based, multidisciplinary approach that integrates education, objective monitoring, self-care, individualized reinforcement, and personalized treatment—all of which are essential components for effective community nursing practice and future research development.

## Conclusions

The “Active Legs” program demonstrated a positive trend in promoting VUs healing in primary care at both 3 and 6 months of follow-up. However, the differences were not statistically significant, most likely due to the smaller-than-expected sample size, which limits the ability to draw definitive conclusions regarding the efficacy of the intervention.

Overall adherence to the intervention was lower than anticipated, with higher participation observed during the initial visits and greater compliance with the home exercise component compared to daily walking. This suboptimal adherence may have influenced the outcomes, underscoring the need for strategies aimed at improving patient engagement and adherence in order to maximize the potential benefits of the program.

Despite these limitations, the findings suggest that physical exercise could serve as an effective adjunctive therapy in the management of venous ulcers, particularly when adherence to the intervention is maintained. Future studies should include larger sample sizes, standardized exercise protocols, and extended follow-up periods to evaluate the long-term effectiveness and clinical applicability of such interventions. Additionally, continued professional training and individualized reinforcement strategies are essential to promote adherence and optimize patient outcomes. Implementing these measures would enable a more robust validation of the program’s effectiveness and support its integration into routine primary care practice.

## Data Availability

There are ethical and legal restrictions on sharing the dataset generated by the study because it contains sensitive clinical information about the people participating in the trial. The Ethics Committee approved this research without considering the option of sharing the data. Therefore, these data will not be publicly available. The Active Legs group may establish future collaborations with other groups based on these data, in which case the principal investigator [BJHA] should be contacted. Each new project based on these data must first be submitted to the Ethics Committee for approval.

## Abbreviations

VU: Venous ulcer
LL: Lower limb
ABI: Ankle-brachial index
CRDe: Electronic data collection notebook
ISCIII: Carlos III Health Institute
